# Don’t Look Back: Post-Disaster Mental Health Benefits of Aging Through Reduced Focus on the Past

**DOI:** 10.1093/geroni/igaf122.347

**Published:** 2025-12-31

**Authors:** JoNell Strough, Andrew Parker, Esha Azhar, Samer Atshan, Ryan Best

**Affiliations:** West Virginia University, Morgantown, West Virginia, United States; RAND Corporation, Pittsburgh, Pennsylvania, United States; West Virginia University, Morgantown, West Virginia, United States; Center for Economic and Social Research, Los Angeles, California, United States; West Virginia University, Morgantown, West Virginia, United States

## Abstract

Disasters negatively impact mental health (NIH, 2022) but some research found that older age was protective (Strough et al., 2023). We used data (n = 936) from six monthly surveys of the Understanding America Study to investigate whether the impact of disasters on depression and anxiety varied across adulthood and if so, whether temporal orientations to the past, present, or future mediated any age differences. Panelists reported whether they had experienced any disaster in the past month (e.g., hurricane, wildfire) and if so, whether they had personally experienced any negative consequence (e.g., evacuation, property loss). Older age was consistently associated with reporting fewer symptoms of depression and anxiety even among those who had experienced a disaster or negative consequence. Depression and anxiety symptoms were significantly greater among individuals who had experienced a negative consequence of a disaster versus not having experienced any disaster, or having experienced a disaster without a negative consequence. Mediation analyses tested present, past, and future temporal orientations as pathways between older age and fewer anxiety and depression symptoms, controlling for income, education, race, and gender. Older age was associated with lesser past and future temporal orientations. Greater temporal orientation toward the past (but not future or present) was significantly associated with increased depression and anxiety symptoms. Age-related reductions in anxiety and depression symptoms were partially explained by a lesser tendency to focus on the past at older ages. Findings are interpreted from the perspective of Carstensen’s (2006) socio-emotional selectivity theory. Implications for policy and practice are discussed.

